# New Method of Preventing Secretion of Milk in Female Breast

**Published:** 1876-04

**Authors:** 


A New Method of Preventing the Secretion of Milk in the Female Breast.—Dr. John Wm. Lane, L. R. C. S., writes to the *Medical Press and Circular:*

I have for more than ten years employed the following method to prevent the secretion of milk in the breasts of women who may have had still-born children, or who, after having nursed their child for a few months, found it necessary to wean it. It is perfectly clean and painless, as far as my experience goes, and as such I beg to recommend it to the notice of my medical brethren.

We will take, for instance, the case where the infant has been born at the full period, but is dead, or dies within a few hours after its birth. The milk makes its appearance in the breasts generally about the second day, sometimes longer, and sometimes it is ready when the child is born, and in the case of still-born children my experience leads me to think that in such cases it makes its appearance earlier than when the child is born alive. My plan consists in taking a piece of emplastrum adhæsivum of about ten inches square, round the corners, cut a hole in the centre for the nipple, then from the centre of each corner make a straight cut toward and within two inches of the centre hole; having now got it ready, let the patient lie on her back, her body being perfectly horizontal; warm the plaster and place it over the breast, then strap one of the lower corners down first, draw the opposite one tightly upward and fix it in its place, then the other lower corner, and lastly the opposite upper one, having drawn it sufficiently tight first; now take a piece of plaster two inches wide and about sixteen or eighteen inches long, and put it on from below and outside the breast, across, close by inside of nipple, and fasten the end over the clavicle; another piece may also be put on in an opposite direction, it being drawn over the shoulder. Of course, in cutting the plaster and strips the size of the breasts must be taken into consideration, there being so much difference in the size of female breasts.

The above plan I always follow when one of my patients wishes to dry the milk, as they usually call it, or when they are compelled to do so either from the death of the child or any other cause. I also am certain that strapping will prevent mammary abscess if resorted to in the earlier stage; I at least have found it to do so in many cases.—*Medical and Surgical Reporter.*

